# “I don’t take for granted that I am doing well today”: a mixed methods study on well-being, impact of cancer, and supportive needs in long-term childhood cancer survivors

**DOI:** 10.1007/s11136-021-03042-6

**Published:** 2021-11-24

**Authors:** Manya Jerina Hendriks, Nathalie Hartmann, Erika Harju, Katharina Roser, Gisela Michel

**Affiliations:** 1 Department of Health Sciences and Medicine, University of Lucerne, Frohburgstrasse 3, PO Box 4466, 6002 Lucerne, Switzerland; 2 Department of Neonatology, Clinical Ethics, University Hospital Zurich, University of Zurich, Zurich, Switzerland

**Keywords:** Childhood cancer survivors, Wellbeing, Health-related quality of life, Impact of cancer, Supportive care needs, Long-term follow-up care, Mixed methods

## Abstract

**Purpose:**

With increasing numbers of childhood cancer survivors (CCS), it is important to identify the impact of cancer and CCS’ needs for support services that can mitigate the long-term impact on psychosocial wellbeing, including health-related quality of life (HRQOL). We aimed (1) to describe survivors’ wellbeing, impact of cancer, and supportive care needs and (2) to determine how socio-demographic or clinical characteristics and impact of cancer relate to survivors’ unmet needs.

**Method:**

In this mixed methods study, a quantitative survey was used to assess HRQOL, psychological distress, impact of cancer, and supportive care needs. Qualitative interviews were conducted to explore the meaning of wellbeing, health, and impact of cancer.

**Results:**

Overall, 69 CCS participated in the survey of which 28 participated in qualitative interviews (aged ≥ 18 years, diagnosed with cancer ≤ 18 years). Few CCS (13%) reported poor physical HRQOL, but almost half reported poor mental HRQOL (49%) and psychological distress (42%). Health was considered to encompass both: physical and emotional aspects of wellbeing. Cancer positively impacted CCS’ ability to care and attitude towards life, whereas relationships and insurance were negatively impacted. Risks for unmet needs increased in CCS with self-reported low health status, late effects, psychological distress, with older age at study or longer time since end of treatment.

**Conclusion:**

In our study, many CCS experienced various psychosocial, psychological and informational unmet needs, indicating that survivors’ needs are currently not duly addressed. Current efforts to provide supportive psychosocial care in Switzerland should be further operationalized to provide adequate support.

**Supplementary Information:**

The online version contains supplementary material available at 10.1007/s11136-021-03042-6.

## Plain English summary

Nowadays, most children can be cured from cancer and become survivors. This means that more survivors experience physical, social, and emotional difficulties after being cured. Still, few survivors get care that goes beyond the medical impact of cancer. The social and emotional wellbeing of survivors is often not addressed by healthcare professionals. Knowing about the unmet needs of survivors can give healthcare professionals important information on how to help survivors and also address their social or emotional problems and/or concerns. In this study, we have explored in-depth the experiences of survivors living in Switzerland on their wellbeing, impact of cancer and unmet needs in care. Results show that the majority of survivors has many unmet needs and indicates the current lack of specific psychosocial care. Hopefully, our findings will encourage healthcare professionals to develop services and provide care tailored to survivors’ unmet needs.

## Introduction

Due to significant advances in therapy, more than 80% of children diagnosed with cancer survive ≥ 5 years [1]. In recent decades, the wellbeing of childhood cancer survivors (CCS) has become a central point of interest for healthcare professionals (HCPs) and researchers [2]. Whilst many long-term medical outcomes are well investigated and documented, few studies have focused on psychosocial late effects of survivors [3]. Psychosocial late effects are common in CCS and may have an impact on their quality of life (QoL) and wellbeing [4]. Late effects not only include medical problems [5], but also worsened social outcomes, such as academic and employment barriers [6, 7], financial and insurance concerns [8, 9], and psychological outcomes like psychological distress, including anxiety and depression [10, 11], and difficulties with family and intimate relationships [12].

Although some CCS receive effective and well-organized medical long-term follow-up care (LTFU), only few survivors encounter comprehensive psychosocial assessments during LTFU despite being recommended as standard of care [13]. Perhaps unsurprisingly, as a result, supportive care needs often remain undetected and undertreated [14–16]. This is worrying, as evidence continues to show a critical subset of survivors at increased risk for psychosocial late effects, psychological distress and poor QoL [11, 17, 18], and warrants special attention from HCPs who work with and care for CCS. One of the vital roles of HCPs is supporting CCS in maintaining a healthy physical and psychosocial wellbeing. In doing so, survivors’ sense of QoL is important as this can differ from HCPs assessment of QoL [19]. Wellbeing is a multifaceted concept that integrates mental and physical health and QoL, while incorporating the presence of positive emotions and moods, the absence of negative emotions, satisfaction with life, fulfillment and positive functioning [20]. With the growing population of CCS, it is important to identify the negative impact of cancer and the related unmet needs faced by CCS to develop appropriate support services and targeted interventions that can mitigate long-term impact on wellbeing [21, 22].

So far, few studies assessed CCS’ unmet needs regarding psychosocial support [14, 21], and most focused on survivors’ information needs [16, 23, 24]. CCS with information needs appear to have more psychological distress and lower health-related quality of life (HRQOL) [22, 24, 25], with HRQOL being understood to “reflect the impact of disease and treatment on disability and daily functioning; it has also been considered to reflect the impact of perceived health on an individual’s ability to live a fulfilling life” [26]. Additionally, several factors can impact needs. The need for information is particularly high in women, those with low household income, those older than 25 years of age, or with a cancer diagnosis at age 5–14 years [23, 24]. So far, there are no published studies among Swiss CCS that extend beyond information needs with just a handful of studies internationally [14, 21]. Most evidence on the impact of cancer and CCS’ needs is based on quantitative studies [14, 19, 21, 24, 27, 28], which by design are limited in-depth of input and knowledge yielded. To increase our understanding of CCS’ experiences and challenges and to present a full picture of the phenomenon, we have selected a mixed methods study design to incorporate the cancer experience as voiced by CCS themselves. This broader understanding of CCS’ functioning will help tailoring interventions based on their needs.

Therefore, this mixed methods study aimed to (1) describe survivors’ wellbeing (including HRQOL and psychological distress), the impact of cancer, and supportive care needs and (2) determine how socio-demographic or clinical characteristics and impact of cancer relate to survivors’ unmet needs.

## Methods

This study applied a mixed methods design [29]; a quantitative survey assessing socio-demographic and clinical characteristics, wellbeing (HRQOL, psychological distress), impact of cancer, and supportive care needs, followed by qualitative interviews expanding on CCS’ wellbeing, health, and impact of cancer.

### Participants and procedure

CCS were identified through Childhood Cancer Switzerland, the umbrella organization of institutions in pediatric oncology. CCS were eligible if they were aged ≥ 18 years, diagnosed with cancer ≤ 18 years of age, completed treatment ≥ 2 years ago, were German or English-speaking Swiss residents.

Childhood Cancer Switzerland invited 132 CCS to participate via e-mail. Additional participants were invited through an open electronic link using SoSciSurvey [30] circulated among Swiss CCS’ networks on social platforms and survivor meetings. The survey could be completed electronically or by phone and with a family member’s help. After two months, a reminder was sent to non-responders. The survey was accessible November 2017–December 2018. Survey participants who were willing to participate in qualitative interviews shared their contact information. We contacted interested participants to schedule interviews at a location of their choice. Interviews were conducted November 2018–February 2019.

The Ethics Committee Northwest and Central Switzerland approved the study (Study-ID: EKNZ 2017-01758). All participants received written study information prior to the survey/interview and CCS participating in the interviews additionally received oral study information. Participants provided informed consent.

### Data collection and measurements

#### Quantitative measurements

Socio-demographic characteristics included sex, age, and educational achievement. Clinical characteristics including diagnosis, age at diagnosis, and treatment (surgery only or chemotherapy, radiation, and bone marrow transplantation) were self-reported by CCS. To avoid treatment categories with very small sample sizes, surgery and chemotherapy were combined. Furthermore, we asked about follow-up care attendance, time since end of treatment, relapse, second malignancy, and health condition. CCS provided information on late effects, including open-ended responses. Responses were categorized into mental, physical, or both types of late effects.

The Short Form-12 (SF-12), a widely used and validated tool [31, 32], assessed HRQOL. The SF-12 yields two summary scores: Physical Component Summary (PCS) and Mental Component Summary (MCS) which are weighted sums of eight subscales. Cronbach’s alpha was adequate to good (Physical Functioning = 0.56, Role Physical = 0.87, Role Emotional = 0.92, Mental Health = 0.82; Bodily Pain, General Health, Vitality, Social Functioning only consist of 1 item each). Raw scores were converted into T-scores (mean = 50, standard deviation = 10) according to age- and sex-stratified norm data from the German Socio Economic Panel [33]. Higher scores indicate better HRQOL. We used cut-off scores based on a population of young Swiss cancer survivors [18], to create binary variables for HRQOL (poor physical health: PCS ≤ 45.5, poor mental health: MCS ≤ 47.5). Health status was assessed using the first question of the SF-12, which is frequently used to measure global self-rated health [34]. Using a five-point Likert scale (1 = ‘excellent’, 2 = ‘very good’, 3 = ‘good’, 4 = ‘fair’, and 5 = ‘poor’), health status was categorized as high (1–3) or low (4–5).

We used the Brief Symptom Inventory-18 (BSI-18), a reliable and valid standardized self-report inventory to assess psychological distress [35], where each item describes a symptom. Participants rate how bothersome a symptom has been during the last week on a five-point Likert scale (0 = ‘not at all’ to 4 = ‘extremely’). Items are summed up to three six-item scales (Somatization, Depression, Anxiety) and a Global Severity Index (GSI) for overall distress [35]. Reliability measured by Cronbach’s alpha was good (Somatization = 0.72, Depression = 0.91, Anxiety = 0.81, GSI = 0.83). Since no normative data are available for Switzerland, we used sex‐specific German normative data to transform raw scores into T-scores. Higher scores indicate higher distress. To identify distressed CCS, we used the standard case rule and the cut-off T-score ≥ 57 proposed by Zabora et al. [36] and previously used in studies in Swiss and American CCS [28, 37]. Individuals with at least two subscales with T ≥ 57, or GSI T ≥ 57 were considered psychologically distressed.

To evaluate the impact of the cancer experience on survivors’ daily living, we adapted the Brief Cancer Impact Assessment (BCIA) [38] to the purpose of our study. The BCIA is a short version of the Impact of Cancer Scale (IOC-CS) [39], which was developed for survivors of adult cancer and has satisfactory validity and reliability [40–43]. It examines perceived negative and positive impact of cancer on eight areas of functioning and activities [43]. We rephrased (education, work, relationships, friendships, family relations) or adapted (insurance, attitudes toward life, and ability to care) the areas to fit our target population. We used a 5-point Likert scale (− 2 and − 1 = ‘negative’, 0 = ’no impact’, + 2 and + 1 = ‘positive’). The areas were grouped into three theoretical domains, 1) *Psychosocial*: insurance, education, and work, 2) *Social*: relationships, friendships, family relations and 3) *Intrapersonal*: attitudes toward life and ability to care. Participants had the option to provide more details via sub-items and open-ended responses.

We assessed met and unmet support needs using an adaption of the questionnaire from Hoven et al. [44], originally based on Stein, Jessop [45], used to identify health care needs (HCNs) of adult CCS. We adapted the 11 items and 4 domains of HCN to focus more specifically on psychosocial aspects of HCN rather than on medical care and care coordination. Hence, respondents completed 6 items covering 3 domains of HCN (*Psychosocial* (work, vocation, education and insurance), *Psychological*, and *Informational Services*) indicating their needs according to the following categories: (1) *used the health service*; (2) *could have needed the health service, which was available, but did not use it;* (3) *had no need for the health service that was available*; (4) *would have needed the health service but it was not available*; or (5) *had no need for the health service.* We considered it important to distinguish between unmet, met needs and no needs rather than no needs vs (some) needs [46]. Therefore, needs were categorized as met (response category 1), no needs (response categories 3 and 5), and unmet (response categories 2 and 4). Similar to Hoven et al., unmet needs were recorded in the *Psychosocial Services* domain if respondents reported unmet needs in at least two items of the domain. Participants were able to further write comments in open-ended responses.

Prior to distribution to participants, the survey was piloted and revised including CCS’ and pediatric oncology experts’ input (see Supplemental Material).

#### Qualitative data collection

Qualitative data included free text responses from the survey and interviews. One-to-one interviews, using an open-ended interview guide, were conducted by the first author, until theoretical saturation was achieved [29]. The interview guide focused on participants’ perceptions of wellbeing, health, and impact of cancer (see Supplemental Material). The interviews occurred at a place most convenient for the participant, were audio recorded, and lasted between 39 and 117 min (average: 89 min).

### Data analysis

We calculated proportions of CCS with poor physical (PCS, SF-12) and mental (MCS, SF-12) health and proportions of CCS reporting negative, none, and positive impact of cancer. We conducted Fisher’s exact tests to analyze associations between type of participants (interview and questionnaire versus questionnaire only) and to determine how socio-demographic or clinical characteristics and impact of cancer relate to (un)met needs. Fisher’s exact test is recommended when more than 20% of cells have expected frequencies < 5 [47, 48]. Analyses were carried out using Stata 16.1 (StataCorp LP, College Station, TX) and *p* ≤ 0.05 was considered statistically significant.

Open-ended responses from the survey and interviews were transcribed, fed into a qualitative data analysis software (ATLAS.ti 8.3), and analyzed. Qualitative data analysis followed the principles of qualitative content analysis [49]. This approach focuses on the importance of context in determining meaning that is data-driven and iterative; i.e. considering previously defined research questions and literature, while allowing categories to emerge out of the data [49]. First, an initial coding scheme was developed based on our research question, interview guide, and reviewed literature (MH, NH). Second, preliminary codes were generated through systematic coding of the data by MH and EH. Third, identified codes were reviewed and the coding scheme was refined with codes that emerged from the data. Discrepancies were resolved through repeated discussion (MH, EH). Finally, the codes were systematically categorized into themes for in-depth analysis.

## Results

### Participant characteristics

Out of 132 eligible participants contacted, 55 survivors returned the questionnaire (42%) and an additional 14 CCS were reached through social platforms resulting in 69 participants (68% were female; for characteristics of participant see Table 1). Twenty-eight participated in the interviews. There were no differences between CCS who did or did not participate in interviews (Table 1).

**Table 1:** Sample description of participating childhood cancer survivors (*n* = 69) and the subgroup of participants who additionally completed an interview (*n* = 28)

	Participants to questionnaire study (*N* = 69)		Subgroup of participants who completed an interview (in addition to the questionnaire) (*N* = 28)		Subgroup of participants to the questionnaire who did not participate in interviews (*N* = 41)	
	*n*	%	*n*	%	*n*	%
*Socio-demographic characteristics*						
Gender						
Male	22	31.9	9	32.1	13	31.7
Female	47	68.1	19	67.9	28	68.3
Age at study						
≤ 25 years	28	40.6	11	39.3	17	41.5
26–30 years	12	17.4	5	17.9	7	17.1
31–35 years	13	18.8	4	14.3	9	22.0
> 35 years	16	23.2	8	28.6	8	19.5
Nationality						
Swiss	61	88.4	24	85.7	37	90.2
Other^b^	8	11.6	4	14.3	4	9.8
Currently in a relationship						
Yes	13	18.8	6	21.4	7	17.1
No	56	81.2	22	78.6	34	82.9
Children						
Yes	12	17.4	6	21.4	6	14.6
No	57	82.6	22	78.6	35	85.4
Education						
Compulsory schooling	5	7.2	4	14.3	1	2.4
Vocational training	36	52.2	13	46.4	23	56.1
Upper secondary	14	20.3	6	21.4	8	19.5
University degree	14	20.3	5	17.9	9	22.0
Employment status						
Employed	51	73.9	24	85.7	27	65.9
Not employed	18	26.1	4	14.3	14	34.1
*Clinical characteristics*						
Diagnosis						
Leukemia	23	33.3	10	35.7	13	31.7
Lymphoma	13	18.8	5	17.9	8	19.5
CNS tumor	10	14.5	3	10.7	7	17.1
Other^c^	23	33.3	10	35.7	13	31.7
Age at diagnosis^d^						
0–5 years	18	26.1	7	25.0	11	26.8
6–11 years	24	34.8	10	35.7	14	34.1
12–17 years	27	39.1	11	39.3	16	39.0
Treatment						
Surgery only or chemotherapy^e^	35	50.7	15	53.6	20	48.8
Radiation^f^	28	40.6	10	35.7	18	43.9
Bone marrow transplantation^g^	6	8.7	3	10.7	3	7.3
Time since end of treatment^d^						
≤ 5 years	8	12.1	3	11.1	5	12.2
6–15 years	22	33.3	8	29.6	14	34.1
16–25 years	22	33.3	6	22.2	16	39.0
> 25 years	14	21.1	10	37.0	4	9.8
Self-reported health status						
High health status	55	79.7	22	78.6	33	80.5
Low health status	14	20.3	6	21.4	8	19.5
HRQOL (SF-12)						
Poor physical health (PCS)	9	13.0	4	14.3	5	12.2
Poor mental health (MCS)	34	49.3	12	42.9	22	53.7
Psychological distress (BSI-18)						
Yes	29	42.0	9	32.1	20	48.8
No	40	58.0	19	67.9	21	51.2
Late effects						
Yes	47	68.1	18	64.3	29	70.7
No	22	31.9	10	35.7	12	29.3
Type of late effects^d^						
Psychological	3	7.1	0	0	3	11.5
Physical	29	69.0	12	75.0	17	65.4
Both	10	23.8	4	25.0	6	23.1
Follow up attendance						
Yes	37	53.6	15	53.6	22	53.7
No, completed	32	46.4	13	46.4	19	46.3
Relapse						
Yes	18	26.1	7	25.0	11	26.8
No	51	73.9	21	75.0	30	73.2
Second malignancy						
Yes	12	17.4	6	21.4	6	14.6
No	57	82.6	22	78.6	35	85.4
*Mean (years, range)*						
Age at study	30.2	17–55	31.4	18–55	29.4	17–51
Age at diagnosis	9.5	0.5–18	9.3	0.5–16	9.6	1–18
Time since end of treatment	17.5	1–38	19.1	1–38	16.4	3–37

*n* number, *CNS* Central Nervous System, *HRQOL* Health-related quality of life, *PCS* Physical Component Summary (SF-12), *MCS* Mental Component Summary (SF-12)

^a^Fisher’s exact test

^b^Includes German, Dutch, Italian, Luxembourg, Serbian, and Philippine nationality

^c^Includes neuroblastoma, renal tumor, bone tumor, soft tissue sarcoma, thyroid cancer, germ cell cancer and histiocytosis

^d^Missing values

^e^Not included radiation

^f^May have included surgery and/or chemotherapy

^g^May have included surgery, chemotherapy and/or radiation

### Wellbeing and HRQOL

One fifth of CCS reported having a low health status (20%). Regarding HRQOL, nine CCS (13%) reported poor physical health (PCS) and 34 (49%) reported poor mental health (MCS). Twenty-nine CCS (42%) were considered psychologically distressed. As a result of experiencing cancer, 23 (33%) survivors changed to a healthier lifestyle and 22 (32%) took fewer risks. When we asked CCS to further elucidate on the meaning of health, they reported that their cancer experience and survivorship had impacted their views on health and wellbeing.

You think differently about life, about health and quality of life: you don't take it for granted. Meaning I don't take for granted that I am doing well today. […] And I know that my feeling well today is not self-evident.—Female survivor, age at diagnosis 0-5, time since end of treatment 16-25 years, without late effects, CCS184.

CCS expressed that being healthy meant living a fulfilled life and participating in normal everyday activities. As such, for most survivor’s health encompassed both, physical and emotional aspects of wellbeing. Being able to consider oneself as “*healthy*” under these terms was equated to having a high QoL.

### Impact of cancer

**Fig. 1:**
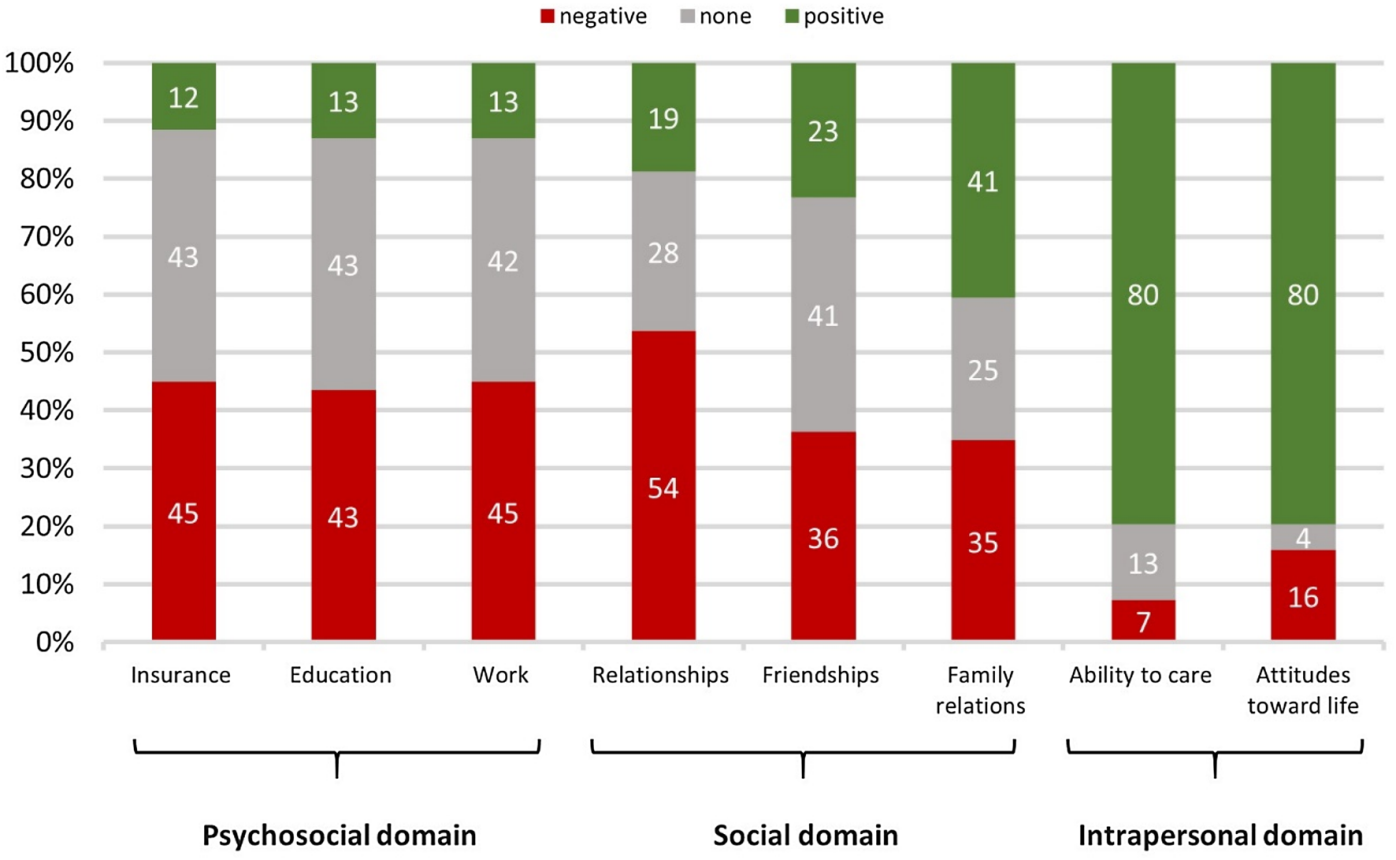
Self-reported impact of cancer for childhood cancer survivors (*n* = 68; proportion of survivors)

In the survey, CCS described that cancer had a diverse impact in various aspects of their life (Fig. 1). Based on the in-depth conversations with CCS, these aspects were grouped into three overarching themes, i.e. the intrapersonal, social, and psychosocial domain (Table 2).

**Table 2:** Selected quotes on impact of cancer

Themes	Sub-themes	Quotes
Intrapersonal domain	Attitude towards life	“So I think I move a little bit, how should I say, more relaxed through life, because I know, well I've seen, how quickly something like this can happen. And that you don’t have to be afraid of everything but just do what you want, of course I'm working towards a goal, I want to achieve it but still I just tell myself that if it doesn’t work out now, then there are other things and a bit of this ease has been given to me through this experience.”—Male survivor, age at diagnosis 6–11 years, ≥ 25 years since end of treatment, with late effects, CCS271
	Ability to care	“More empathetic, but I also have the feeling that I quickly lose sight of the context and thus ‘get lost in somethings’ more quickly.”—Female survivor, age at diagnosis 12–17 years, 11–15 since end of treatment, with late effects CCS177
Social domain	Relationships	“Relationships that is just more difficult. I would have liked to have one already but when it comes to it it’s still difficult. I honestly don’t know exactly why not and where the problem lies. It is difficult. It’s just, I'm having a hard time. When I think maybe it will work now, then the problem happened with my shoulder and, yes, and then the self-confidence is not so high anymore. And I think maybe that is part of it.”—Female survivor, age at diagnosis 12–17 years, 11–15 years since end of treatment, with late effects CCS187
	Friendships	“It showed me which friends really stood by me. It brought me closer to a lot of people, but I also lost a lot, but I feel this is positive because now I know exactly whom I can trust and who stands by me in difficult times.” Female survivor, age at diagnosis 12–17 years, ≤ 5 years since end of treatment, with late effects, CCS181
	Family relations	“Because of the disease, our family became very close. We know that we can rely on each other for 100 percent (…). But my grandmother always treated me like a helpless, dependent child. To this day my mother still protects me more than my siblings and accepts my independence much less.”—Male survivor, age at diagnosis 6–11 years, ≥ 25 years since end of treatment, with late effects, CCS191
Psychosocial domain	Insurance	“I got denied supplementary [health] insurances for something that is out of my control. I was just a child.”—Female survivor, age at diagnosis 12–17 years, 11–15 years since end of treatment, with late effects CCS182
	Education	“I appreciated school more than before the illness. Although I tried to learn what I missed… many aspirations did not align with the "wish study". So I decided to take an apprenticeship. Could not be more grateful for this decision”—Male survivor, age at diagnosis 12–17 years, 11–15 years since end of treatment, with late effects CCS213
	Work	“In the beginning I had to explain to each employee again and again what I had and why I was handicapped [pulmonary problems, osteoarthritis, and peripheral neuropathy].”—Female survivor, 12–17 years, ≤ 5 years since end of treatment, with late effects, CCS181

### Intrapersonal impact

CCS’ attitude towards life (80%) and ability to care (80%) had been positively impacted. A large majority of CCS reported becoming more empathic (81%), whereas few participants considered themselves to have become more distant towards people (17%). In the interviews, CCS further described appreciating the beautiful moments in life as this was not a given and reported on the “*finiteness of life*”. Specifically, CCS frequently mentioned they “*valued life more*” and lived more consciously. They aimed to enjoy the present and “*live in the now*”. CCS’ considered that their experiences gave them the unique possibility to think and reflect about their life goals and envision a different path than they had previously imagined.

### Social impact

Over half of CCS reported cancer had negatively impacted their relationships in general (54%) Relationships with friends were negatively impacted for 36%, and 35% reported negative impact on relations with family members. CCS often started a relationship only later in life (48%) and many were concerned about having children (48%). Some CCS were burdened by experiencing infertility (19%). Regarding friendships, almost half of CCS (42%) reported losing a friend due to their cancer experience and 31% considered it more difficult to meet new people. On the other hand, some CCS gained new friendships because of their disease (30%). Half of CCS (52%) felt they were a burden to their family and about one third of CCS (29%) felt their parents were overprotective. In the interviews, CCS indicated some disadvantages could also be considered advantages. Although many survivors mentioned looking for a partner causing distress, others added that their experience had given them a better idea of what an ideal partner should be like. Some CCS described “*the struggle of accepting their body*” and “*fear for intimacy*”. Fear of intimacy was closely related to a general difficulty in trusting, which applied for partnerships and also friendships. CCS described how they overcome their issues of distrust in building meaningful relations with partners and friends. Most CCS have a small circle of friends due to this constant evaluation on whether to trust someone. Overprotection was the only downside of familial support and was often related to independence.

### Psychosocial impact

Many CCS reported cancer had negatively affected their education (43%). Only 7% dropped out of school, but 23% changed schools because their school performance was affected (15%) or they had problems with classmates (10%). In addition, 26% of CCS experienced “mobbing” from other classmates. For 45% of CCS a negative impact on work was reported. CCS reported restraints in their work life due to bodily performance (39%) or psychological problems (25%). For insurance matters, 45% of CCS felt their disease had negatively impacted this aspect of their life. Some CCS indicated they were ineligible for supplementary health insurance (21%) and their late effects went unacknowledged (25%). During interviews, many survivors mentioned they felt excluded and/or misunderstood by their classmates because they were not well informed by teachers. Such misunderstandings were also mentioned by some working CCS, who thought missing appointments due to LTFU made them seem unreliable to their boss and/or colleagues.

### (Un)met needs for support

**Fig. 2:**
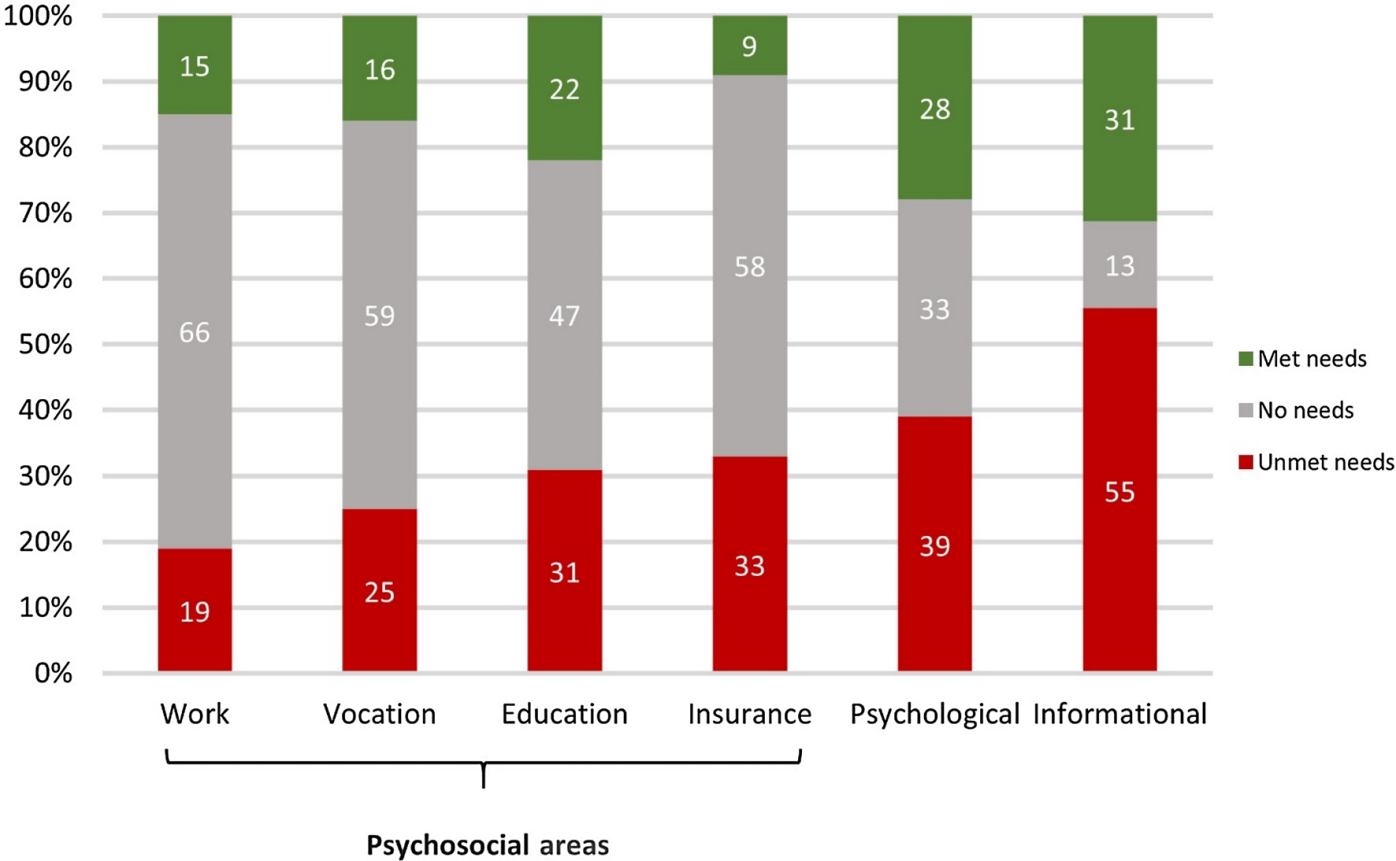
Self-reported met and unmet needs for psychosocial support for childhood cancer survivors (*n* = 69; proportion of survivors)

Overall, a majority of CCS (81%) reported at least one unmet supportive care need (mean of two per survivor). The most common unmet needs involved informational support (55%), psychological support (39%), support with insurance (33%), and educational support (31%, Fig. 2).

We found several associations between CCS’ unmet psychosocial, psychological and information needs and socio-demographic, clinical characteristics and impact of cancer (Table 3). Overall, no associations existed regarding poor HRQOL or follow-up care. CCS who reported a low health status (*p* = 0.008) and those reporting psychological and physical late effects (*p* = 0.034) were more likely to report psychosocial unmet needs. CCS who reported a negative impact in the psychosocial domain were more likely to have unmet psychosocial needs (*p* = 0.039). With regards to psychological needs, older CCS at study (*p* = 0.021) or aged 6–11 years at diagnosis (*p* = 0.005) were more likely to report an unmet need. CCS who reported a negative impact in the intrapersonal domain (*p* = 0.015) and with longer time since end of treatment (*p* = 0.006) were more likely to have psychological unmet needs. CCS who were older at study (*p* = 0.048), longer time since end of treatment (*p* = 0.043), and CCS with psychological distress (*p* = 0.050) were more likely to report unmet informational needs.

**Table 3:** Associations between socio-demographic characteristics, clinical characteristics, and impact of cancer and unmet needs of childhood cancer survivors

	Unmet needs									
	Total	Psychosocial			Psychological			Informational		
		*n*	%	*p*^a^	*n*	%	*p*^a^	*n*	%	*p*^a^
*Socio-demographic characteristics*										
Gender										
Male	22	9	40.9	0.284	11	50.0	0.186	12	54.6	0.999
Female	47	13	27.6		15	31.9		25	53.2	
Age at study										
≤ 25 years	28	10	35.7	0.090	7	25.0	**0.021**	10	35.7	**0.048**
26–30 years	12	3	25.0		2	16.7		6	50.0	
31–35 years	13	1	7.7		7	53.9		9	69.2	
> 35 years	16	8	50.0		10	62.5		12	75.0	
Nationality										
Swiss	61	20	32.8	0.999	24	39.3	0.701	32	52.5	0.716
Other^b^	8	2	25.0		2	25.0		5	62.5	
Relationship										
Yes	13	4	30.7	0.999	6	46.2	0.535	8	61.5	0.556
No	56	18	32.1		20	35.7		29	51.8	
Children										
Yes	12	4	33.3	0.999	6	50.0	0.347	7	58.3	0.761
No	57	18	31.6		20	35.1		30	52.6	
Education										
Compulsory schooling	5	0	0	0.292	2	40.0	0.324	3	60.0	0.999
Vocational training	36	14	38.9		10	27.8		19	52.8	
Upper secondary	14	3	21.4		7	50.0		8	57.1	
University degree	14	5	35.7		7	50.0		7	50.0	
Employment status										
Employed	51	14	27.5	0.241	19	37.3	0.999	28	54.9	0.787
Not employed	18	8	44.4		7	38.9		9	50	
*Clinical characteristics*										
Diagnosis										
Leukemia	23	10	43.5	0.338	10	43.5	0.692	12	52.2	0.865
Lymphoma	13	4	30.8		5	38.5		8	61.5	
CNS tumour	10	1	10		2	20.0		6	60.0	
Other^c^	23	7	30.4		9	39.1		11	47.8	
Age at diagnosis^d^										
0–5 years	18	4	22.2	0.076	6	33.3	**0.005**	7	38.9	0.328
6–11 years	24	12	50.0		15	62.5		14	58.3	
12–17 years	27	6	22.2		5	18.5		16	59.3	
Treatment										
Surgery only or chemotherapy^e^	35	12	34.3	0.927	12	34.3	0.758	15	42.9	0.139
Radiation^f^	28	8	28.6		11	39.3		19	67.9	
Bone marrow transplantation^g^	6	2	33.3		3	50.0		3	50.0	
Time since end of treatment^d^										
≤ 5 years	8	2	25.0	0.569	0	0	**0.006**	4	50.0	**0.043**
6–15 years	22	8	36.4		7	31.8		10	45.5	
16–25 years	22	5	22.7		9	40.9		9	40.9	
> 25 years	14	6	42.9		10	71.4		12	85.7	
Self-reported health status										
High health status	55	13	23.6	**0.008**	20	36.4	0.760	27	49.1	0.229
Low health status	14	9	64.3		6	42.9		10	71.4	
Poor physical health (PCS)										
Yes	9	3	33.3	0.999	2	22.2	0.466	5	55.6	0.999
No	60	19	31.7		24	40.0		32	55.3	
Poor mental health (MCS)										
Yes	34	14	41.2	0.126	15	44.1	0.326	20	58.8	0.472
No	35	8	22.9		11	31.4		17	48.6	
Psychological distress										
Yes	29	12	41.4	0.193	10	34.5	0.802	20	68.9	**0.050**
No	40	10	25.0		16	40.0		17	42.5	
Late effects										
Yes	47	17	36.2	0.406	17	36.2	0.792	25	53.2	0.999
No	22	5	22.7		9	40.9		12	54.6	
Type of late effects										
Psychological	3	0	0	**0.034**	0	0	0.324	2	66.7	0.676
Physical	29	9	31.0		10	34.5		15	51.7	
Both	10	7	70.0		5	50.0		7	70.0	
Follow up attendance										
Yes	37	12	32.4	0.999	13	35.1	0.804	20	54.1	0.999
No	32	10	31.2		13	40.6		17	53.1	
Relapse										
Yes	18	5	27.8	0.774	8	44.4	0.575	12	66.7	0.273
No	51	17	33.3		18	35.3		25	49.0	
Second malignancy										
Yes	12	6	50.0	0.177	5	41.7	0.754	8	66.7	0.359
No	57	16	28.1		21	36.8		29	50.8	
*Impact of cancer*										
Negative impact of cancer in psychosocial domain										
Yes	51	20	39.2	**0.039**	20	39.2	0.780	29	56.9	0.418
No	18	2	11.1		6	33.3		8	44.4	
Negative impact of cancer in social domain										
Yes	52	17	32.7	0.999	19	36.5	0.778	29	55.8	0.584
No	17	5	29.4		7	41.2		8	47.1	
Negative impact of cancer in intrapersonal domain										
Yes	15	7	46.7	0.213	10	66.7	**0.015**	10	66.7	0.381
No	54	15	27.8		16	29.6		27	50.0	

Boldface = significant (*p* ≤ 0.05)

*n* number, *CNS* Central Nervous System, *PCS* Physical Component Summary, *MCS* Mental Component Summary

^a^Fisher exact test

^b^Includes German, Dutch, Italian, Luxembourg, Serbian, and Philippine nationality

^c^Includes neuroblastoma, renal tumor, bone tumor, soft tissue sarcoma, thyroid cancer, germ cell cancer and histiocytosis

^d^Missing values

^e^Not included radiation

^f^May have included surgery and/or chemotherapy

^g^May have included surgery, chemotherapy and/or radiation

## Discussion

This study provided information on wellbeing and HRQOL of CCS and illustrated the impact of cancer on various psychosocial areas. Overall, we found a need for improved psychosocial and psychological support among long-term CCS. Despite the overall good physical HRQOL, a subgroup of CCS reported poor mental HRQOL. This is in accordance with previous research [17, 50, 51]. On average CCS have *less* psychological distress than their peers, but the proportion of CCS *at risk* for high psychological distress is disproportionally large [11]. In comparison to other studies, the proportion of CCS with psychological distress in our study was higher [28, 52]. Recent studies have indicated that participants with higher levels of emotional distress are more likely to have poor HRQOL [53, 54].

Moreover, the interviews provided valuable additional information on CCS’ understanding of health, wellbeing and the impact of cancer. CCS elucidated the importance of considering health and wellbeing as encompassing both, physical and psychological aspects. This further underlines the need for HCPs to address medical and emotional needs and monitor psychological distress in CCS during LTFU [11, 50, 55]. In addition, most CCS presented a nuanced description of the impact of cancer often describing it as a “double-edged sword” that includes the struggles alongside the lessons learned [10, 56]. CCS’ ability to care and their attitude towards life were especially reported as positively impacted. Other studies describe such positive effects of the cancer experience [10, 19, 56, 57]. A Swiss study on posttraumatic growth after childhood cancer observed positive changes in relating to others and appreciation of life [58].

Exploring and mitigating the unmet needs of survivors in LTFU is important not only for a subset but for all CCS. It is interesting to note that age seems to impact the unmet needs. With increasing age and time since diagnosis, psychological and informational unmet needs were more likely. This indicates that CCS require information not only after diagnosis and end of treatment, but information needs to be adapted to age and developmental stage and provided in the long-term, along with psychological support [21]. This further underlines the specific challenges faced by *childhood* cancer survivors. Compared to adult cancer survivors, CCS in adulthood have longer experience with the psychological, psychosocial and physical challenges that come with the aftermath of cancer. It is also important to investigate the different age groups and needs among CCS (i.e. children, young adults and older adults) to understand specific vulnerabilities and provide targeted interventions. One recent study has shown that young adult CCS appear to be vulnerable to psychosocial difficulties [27]. Similarly, our study shows that older adult CCS are more prone to experience psychological or informational needs. Our findings further confirm that unmet informational needs are associated with higher levels of psychological distress [25].

Our findings show that regardless of LTFU attendance, CCS’ continue to experience psychosocial, psychological and informational unmet needs. A possible explanation for this might be that although all Swiss pediatric oncology clinics currently offer LTFU for at least 5–10 years, only around one in every four survivors aged > 20 years attends LTFU after their discharge from Pediatric Oncology [59]. Hence, currently LTFU might fail to identify and address CCS’ needs for supportive psychosocial care long into their survivorship. Recently, more attention is being given to LTFU in Switzerland and the first interdisciplinary LTFU clinics for adult CCS are established [60].

Our study has limitations, including a small sample size and a response rate of 48%. However, the response rate is common for this study population [61–64]. Additionally, our sample was not population-based and survivors contacted through Childhood Cancer Switzerland may be more engaged and better informed than other survivors [61]. Therefore, the proportion of unmet needs might either be underestimated or overestimated. This might have resulted in the high number of CCS with psychological distress in our study. In addition, survivors with needs might be more likely to participate. Our results are therefore not generalizable to all Swiss CCS. Our sample has a higher mean age at diagnosis and fewer CCS with brain tumors than other studies [65]. Furthermore, despite unmet needs, CCS might have received psychosocial support, but forgotten about it, or support was offered to parents [67].

Our study also has some strengths. A recognized method to increase participation in vulnerable populations was applied in which CCS could use online or phone-based surveys (one participant did so) [66]. In the survey, CCS’ reflections on the impact of cancer were limited to post-cancer experiences. In the interviews, we therefore sought to explore CCS’ perceptions on the impact of cancer in a broader way. CCS had the opportunity to give a nuanced picture of the impact. In addition, qualitative interviews took place at a location most comfortable to participants. We believe the mixed methods design of our study is a strength as it overcomes the limits of using only a quantitative or qualitative design. The mixed methods study design allowed for a detailed analysis of the impact of cancer on CCS’ lives, which enhances the integrity of the findings. Another strength of our study was the use of the validated SF-12 and BSI-18 questionnaires with specifically calculated cut-offs for CCS.

Despite calls for psychosocial follow-up as a standard of care during survivorship and concrete efforts to improve and include psychosocial services in LTFU [13, 68], our study indicates that the supportive care needs of Swiss CCS are not duly addressed. A large majority of survivors reports at least one unmet need, and CCS with a low health status, psychological and physical late effects, or psychological distress are more likely to experience unmet needs. Especially older adult CCS would benefit from LTFU. Addressing their supportive care needs is a crucial step to improving LTFU. Hence, current efforts to provide supportive psychosocial care in LTFU clinics in Switzerland should be further promoted, including consistently assessing the supportive care needs of CCS. Moreover, psychosocial follow-up care should not just be a recommendation; it is essential for a considerable subset of CCS and likely benefits all. Regular psychosocial screening, provision of age-adapted information on disease and late effects, and support with education and insurance may help to further improve wellbeing and QoL in survivors of childhood cancer.

## Supplementary Information

Below is the link to the electronic supplementary material.

Supplementary file1 (DOCX 24 KB)

Supplementary file2 (DOCX 111 KB)

Supplementary file3 (DOCX 121 KB)

## Data Availability

Anonymized data analyzed during this study may be provided by Dr. Hendriks upon written request.

## Code availability

Dr. Hendriks can be contacted to request an anonymized copy of data used to generate results included in this manuscript.
