# Supplementary material for: “I don’t take for granted that I am doing well today”: a mixed methods study on well-being, impact of cancer, and supportive needs in long-term childhood cancer survivors
Source: Qual Life Res. 2021 Nov 24;31(5):1483–97. doi: 10.1007/s11136-021-03042-6 (PMC9023419; doi:10.1007/s11136-021-03042-6)
Supplement: Supplementary file 1 — Supplementary file1 (DOCX 24 KB) [file 11136_2021_3042_MOESM1_ESM.docx]

| **Medical history and general information** | |
| --- | --- |
| *General socio-demographic data (to be filled in or taken from the questionnaire)*  Participants are invited to narrate the whole arc of their cancer story (process from diagnosis, through treatment, to survivorship and now). They can start at any timepoint, i.e. where their story “started”.  Verbal and non-verbal encouragement to continue with the narrative ("what happened next?", "why do you remember this specific moment?")  The questions below are used to ask specific questions after the narrative. |  |

| **Wellbeing and Health** | |
| --- | --- |
| How would you describe your well-being?  What does the notion of health mean to you? |  |

| **Impact of cancer** | |  |
| --- | --- | --- |
| Participants are invited to talk about the impact of cancer on the life course (positive and negative aspects).   - Verbal and non-verbal encouragement to continue with the story ('what happened then?', 'why do you remember this specific moment?')   Can you say something about whether or not cancer has impacted your life? e.g.:   - School - Work - Families - Friends - Relations |  | |

| **Concluding questions** | |
| --- | --- |
| Questions / additions?  *Thank you so much for participating in this interview.* |  |
