# Supplementary material for: “I don’t take for granted that I am doing well today”: a mixed methods study on well-being, impact of cancer, and supportive needs in long-term childhood cancer survivors
Source: Qual Life Res. 2021 Nov 24;31(5):1483–97. doi: 10.1007/s11136-021-03042-6 (PMC9023419; doi:10.1007/s11136-021-03042-6)
Supplement: Supplementary file 2 — Supplementary file2 (DOCX 111 KB) [file 11136_2021_3042_MOESM2_ESM.docx]

**Supplemental Material**

**Supplemental Figure 1** Conceptual Framework for Impact of Cancer^i^


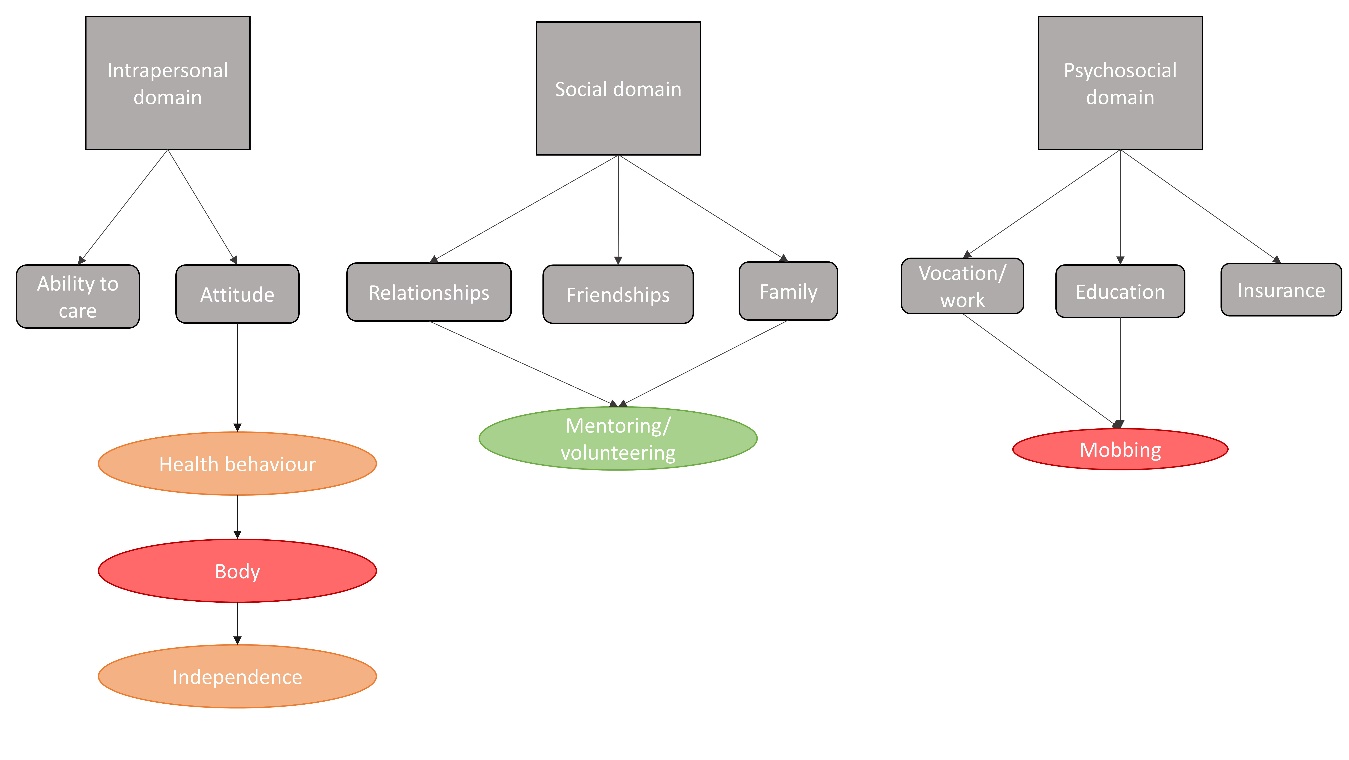


^i^Colour represents the positivity (green), partly positive/negative (orange) or negativity (red) of survivors experience
