# Supplementary material for: “I don’t take for granted that I am doing well today”: a mixed methods study on well-being, impact of cancer, and supportive needs in long-term childhood cancer survivors
Source: Qual Life Res. 2021 Nov 24;31(5):1483–97. doi: 10.1007/s11136-021-03042-6 (PMC9023419; doi:10.1007/s11136-021-03042-6)
Supplement: Supplementary file 3 — Supplementary file3 (DOCX 121 KB) [file 11136_2021_3042_MOESM3_ESM.docx]

Swiss Study to Survey Psychosocial Needs of Former Childhood Cancer Patients

Influence of cancer on attitude to life

*For the statements below, indicate the extent to which cancer has affected you* ***positively*** *or* ***negatively in*** *these areas.*

*Do you* ***feel*** *that areas of your life have been* ***different because of cancer*** *than they would have been if you had not had the disease?*

*How did you* ***feel about the impact of*** *cancer on the above areas* ***after treatment for*** *childhood cancer?*

1. What impact has cancer had on your ability to empathize with and/or be there for others (e.g., spouse, children, parents, friends)?

| Very negative influence | negative influence | no influence | positive influence | Very positive influence |
| --- | --- | --- | --- | --- |
| ☐ | ☐ | ☐ | ☐ | ☐ |

- 1. What influence did the cancer have (multiple answers possible)?

☐ I am more empathetic because of my cancer.

☐ I am distancing myself more from other people because of my cancer.

☐ other (positive or negative) consequences:


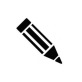


………………………………………………………………………………………………

………………………………………………………………………………………………

………………………………………………………………………………………………

1. What impact did the cancer have on your education (e.g., support you could have used and/or limitations in achieving goals)?

| Very negative influence | negative influence | no influence | positive influence | Very positive influence |
| --- | --- | --- | --- | --- |
| ☐ | ☐ | ☐ | ☐ | ☐ |

- 1. What influence did the cancer have (multiple answers possible)?

☐ Absences due to my cancer treatment

☐ Impaired performance/ concentration caused by my cancer

☐ I was bullied / teased

☐ I had to repeat (a) class(es)

☐I dropped out of school

☐ Change of school due to:

☐ Limitation of benefits due to my cancer

☐ Relocation due to my cancer illness

☐ Problems with classmates due to my cancer disease

☐ Lack of handicapped-accessible premises, which became necessary due to my cancer illness

☐ I got better at school because of my cancer

☐ other (positive or negative) consequences:


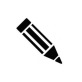


………………………………………………………………………………………………

………………………………………………………………………………………………

………………………………………………………………………………………………

1. What impact did the cancer have on your family relationships (think about changes in your relationship with your parents and/or siblings due to the cancer)?

| Very negative influence | negative influence | no influence | positive influence | Very positive influence |
| --- | --- | --- | --- | --- |
| ☐ | ☐ | ☐ | ☐ | ☐ |

- 1. What areas has cancer affected (multiple answers possible)?

Parents and siblings:

I have a closer relationship with

☐ Parents

☐ Siblings,

through my cancer

I have a more distanced relationship with

☐ Parents

☐ Siblings,

through my cancer

☐ My parents are overprotective because of my cancer.

☐ My parents separated because of my cancer.

Guilt:

☐ I envy my healthy siblings.

☐ I feel I have been a burden to my family because of my cancer.

☐ I feel like my parents feel guilty about my cancer.

Self-employment:

☐ I later detached from my parents/moved out later than peers due to my cancer.

☐ I moved out earlier than my peers because of my cancer.

☐ I need care/support from my parents due to my cancer.

☐ other (positive or negative) consequences:


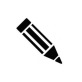


………………………………………………………………………………………………

………………………………………………………………………………………………

………………………………………………………………………………………………

1. What impact did cancer have on your relationship ideas and desires (e.g., finding a partner, getting married, having children, and related fears or concerns)?

| Very negative influence | negative influence | no influence | positive influence | Very positive influence |
| --- | --- | --- | --- | --- |
| ☐ | ☐ | ☐ | ☐ | ☐ |

- 1. Which area has the cancer affected (multiple answers possible)?

Partnership:

☐ I had more trouble finding a partner or a partner later than my peers.

☐ I used to have a partner as a peer.

☐ I can't/won't enter into a partnership because of my cancer.

Have children:

☐ I have more worries or fears about having children than peers because of my cancer.

☐ I can't have children because of my cancer.

☐ other (positive or negative) consequences:


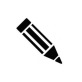


………………………………………………………………………………………………

………………………………………………………………………………………………

………………………………………………………………………………………………

1. What impact did cancer have on your circle of friends (think about experiences you had with your circle of friends because of cancer)?

| Very negative influence | negative influence | no influence | positive influence | Very positive influence |
| --- | --- | --- | --- | --- |
| ☐ | ☐ | ☐ | ☐ | ☐ |

- 1. What influence did the cancer have (multiple answers possible)?

Number of friends:

☐ I have lost friends through my cancer.

☐ I have made friends through my cancer.

I have a majority of friends who also have/had cancer.

Meet new people:

☐ I find it harder to meet new people than people my own age.

☐ I find it easier to meet new people than people my own age.

☐ other (positive or negative) consequences:


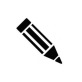


………………………………………………………………………………………………

………………………………………………………………………………………………

………………………………………………………………………………………………

1. What impact did the cancer have on your daily work/career (think about your potential that you are (not) able to realize and/or your satisfaction with your job, equal opportunities)?

| Very negative influence | negative influence | no influence | positive influence | Very positive influence |
| --- | --- | --- | --- | --- |
| ☐ | ☐ | ☐ | ☐ | ☐ |

- 1. What influence did the cancer have (multiple answers possible)?

☐ I have physical performance limitations due to my cancer.

☐ I have mental performance limitations due to my cancer.

☐ I can't do the job I would have liked to do.

☐ other (positive or negative) consequences:


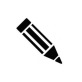


………………………………………………………………………………………………

………………………………………………………………………………………………

………………………………………………………………………………………………

1. What influence did the cancer have on your satisfaction with your insurance situation (health insurance, IV; think about experiences you had after the treatment of the cancer)?

| Very dissatisfied | dissatisfied | no influence | satisfied | very satisfied |
| --- | --- | --- | --- | --- |
| ☐ | ☐ | ☐ | ☐ | ☐ |

- 1. What influence did the cancer have (multiple answers possible)?

☐ I cannot have supplementary health insurance.

☐ I have unrecognized late effects (e.g., damaged teeth from chemotherapy).

☐ I have not been paid (reimbursed) for (a) treatment(s).

☐ I have additional costs due to cancer which are not paid (reimbursed).

☐ other (positive or negative) consequences:


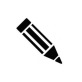


………………………………………………………………………………………………

………………………………………………………………………………………………

………………………………………………………………………………………………

1. What influence did the cancer have on your attitude towards life?

| Very negative influence | negative influence | no influence | positive influence | Very positive influence |
| --- | --- | --- | --- | --- |
| ☐ | ☐ | ☐ | ☐ | ☐ |

- 1. What influence did the cancer have (multiple answers possible)?

☐ I have a healthier lifestyle due to cancer.

Risk Taking:

☐ I take fewer risks because of my cancer.

☐ I take more risks because of my cancer.

Finding Meaning:

☐ I appreciate life more because of my cancer.

☐ I see less meaning in life because of my cancer.

☐ other (positive or negative) consequences:


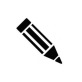


………………………………………………………………………………………………

………………………………………………………………………………………………

…………… …………………………………………………………………………………

Psychosocial support

*For the following offerings, think about the extent to which you would have used or desired them. Refer to the time after therapy. Briefly describe your experience.*

Support areas 10. Can you briefly describe your experience?

| Psychological support  ☐ used the health service  ☐ could have needed the health service, which was available, but did not use it  ☐ had no need for the health service that was available  ☐ would have needed the health service but it was not available  ☐ had no need for the health service | 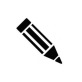 |
| --- | --- |
| Information on cancer and possible late effects  ☐ used the health service  ☐ could have needed the health service, which was available, but did not use it  ☐ had no need for the health service that was available  ☐ would have needed the health service but it was not available  ☐ had no need for the health service | 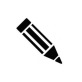 |
| Support during school time/in school  ☐ used the health service  ☐ could have needed the health service, which was available, but did not use it  ☐ had no need for the health service that was available  ☐ would have needed the health service but it was not available  ☐ had no need for the health service | 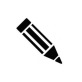 |
| Support in the choice of profession  ☐ used the health service  ☐ could have needed the health service, which was available, but did not use it  ☐ had no need for the health service that was available  ☐ would have needed the health service but it was not available  ☐ had no need for the health service | 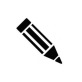 |
| Support with work (part-time job, IV, job search, etc.)  ☐ used the health service  ☐ could have needed the health service, which was available, but did not use it  ☐ had no need for the health service that was available  ☐ would have needed the health service but it was not available  ☐ had no need for the health service | 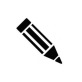 |
| Support with insurances (e.g. health insurance, IV)  ☐ used the health service  ☐ could have needed the health service, which was available, but did not use it  ☐ had no need for the health service that was available  ☐ would have needed the health service but it was not available  ☐ had no need for the health service | 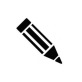 |
| Support in other areas  (partnership, finding a family doctor, etc.)  ☐ used the health service  ☐ could have needed the health service, which was available, but did not use it  ☐ had no need for the health service that was available  ☐ would have needed the health service but it was not available  ☐ had no need for the health service | 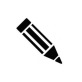 |
| 1. Are there other areas where you have had, or would have liked to have had, support? Describe them briefly. | |

General support questions:

12. Are you dependent on aids or home care services (wheelchair, prosthesis, Spitex, or similar)?

☐ Yes, to the following tools:


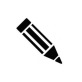


………………………………………………………………………………………………

☐ No

- 1. Are you having trouble getting these tools?

☐ Yes

☐ No

If yes, what problems?


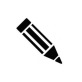


………………………………………………………………………………………………

………………………………………………………………………………………………

………………………………………………………………………………………………

1. Can you talk to someone about your cancer?


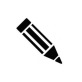


☐ Yes, with:

☐ No

- 1. **If no: Would you have liked to have such a person?**

☐ Yes

☐ No

1. Are you part of a group for former childhood cancer patients, e.g. on Facebook, Twitter, WhatsApp or patient/support group?

☐ Yes

☐ No

- 1. **If no: Would you have liked to see such a group?**

☐ Yes

☐ No

How are you?

*The next section is about assessing your health. For each of the following questions, please check the box of the answer option that best applies to you.*

1. How would you describe your health in general?

Excellent ☐ 1

Very good ☐ 2

Good ☐ 3

Fair ☐ 4

Poor ☐ 5

1. The following questions describe activities you might perform on a normal day. Are you limited in these activities by your current health condition? If yes, to what extent?

|  | Yes, strongly limited | Yes, somewhat limited | No, not restricted at all |
| --- | --- | --- | --- |
| Medium-difficulty activities, e.g., moving a table, vacuuming, bowling, playing golf | ☐ 1 | ☐ 2 | ☐ 3 |
| Climb several flights of stairs | ☐ 1 | ☐ 2 | ☐ 3 |

1. In the past 4 weeks, how often have you had the following difficulties at work or other daily activities at work or at home because of your physical health?

|  | Always | Mostly | Sometimes | Rare | Never |
| --- | --- | --- | --- | --- | --- |
| I could not work as long as usual | ☐ 1 | ☐ 2 | ☐ 3 | ☐ 4 | ☐ 5 |
| I could only do certain things | ☐ 1 | ☐ 2 | ☐ 3 | ☐ 4 | ☐ 5 |

1. In the past 4 weeks, how often did you experience the following difficulties at work or other daily activities at work or at home due to mental health problems (e.g., feeling down or anxious)?

|  | Always | Mostly | Sometimes | Rare | Never |
| --- | --- | --- | --- | --- | --- |
| I have managed less than I wanted | ☐ 1 | ☐ 2 | ☐ 3 | ☐ 4 | ☐ 5 |
| I could not work as carefully as usual | ☐ 1 | ☐ 2 | ☐ 3 | ☐ 4 | ☐ 5 |

1. In the past 4 weeks, to what extent has pain interfered with you performing your daily activities at home or at work?

Not at all ☐ 1

Something ☐ 2

Moderate ☐ 3

Quite ☐ 4

Very ☐ 5

*The next questions are about how you feel and how you have been doing over the past 4 weeks.*

1. Please check the number in each row that most closely matches how you feel.

How often in the past 4 weeks have you been....

|  | Always | Mostly | Sometimes | Rare | Never |
| --- | --- | --- | --- | --- | --- |
| calm and serene? | ☐ 1 | ☐ 2 | ☐ 3 | ☐ 4 | ☐ 5 |
| full of energy? | ☐ 1 | ☐ 2 | ☐ 3 | ☐ 4 | ☐ 5 |
| discouraged and sad? | ☐ 1 | ☐ 2 | ☐ 3 | ☐ 4 | ☐ 5 |

1. In the past 4 weeks, how often have your physical health or mental health problems interfered with your contacts with other people (visits to friends, relatives, etc.)?

Always ☐ 1

Mostly ☐ 2

Sometimes ☐ 3

Rare ☐ 4

Never ☐ 5

Your condition

| 1. *Below is a list of problems and complaints that you can sometimes have. Please read each question carefully and decide how much you have been bothered or distressed by these complaints* ***during the last 7 days****. Some problems or complaints occur very rarely. Nevertheless, please comment on each statement.* | | | | | | | | |
| --- | --- | --- | --- | --- | --- | --- | --- | --- |
|  |  | | | | | | |  |
| **How much have you suffered in the last 7 days from** | | | | | | | |  |
|  |  | | Not at all | A little | quite | strong | Very strong | |
|  | 1 | Faintness or dizziness | 1 | 2 | 3 | 4 | 5 | |
|  | 2 | Feeling no interest in things | 1 | 2 | 3 | 4 | 5 | |
|  | 3 | Nervousness or shakiness inside | 1 | 2 | 3 | 4 | 5 | |
|  | 4 | Pains in heart or chest | 1 | 2 | 3 | 4 | 5 | |
|  | 5 | Feeling lonely | 1 | 2 | 3 | 4 | 5 | |
|  | 6 | Feeling tense or keyed up | 1 | 2 | 3 | 4 | 5 | |
|  | 7 | Nausea or upset stomach | 1 | 2 | 3 | 4 | 5 | |
|  | 8 | Feeling blue | 1 | 2 | 3 | 4 | 5 | |
|  | 9 | Suddenly scared for no reason | 1 | 2 | 3 | 4 | 5 | |
|  | 10 | Trouble getting your breath | 1 | 2 | 3 | 4 | 5 | |
|  | 11 | Feelings of worthlessness | 1 | 2 | 3 | 4 | 5 | |
|  | 12 | Spells of terror or panic | 1 | 2 | 3 | 4 | 5 | |
|  | 13 | Numbness or tingling in parts of your body | 1 | 2 | 3 | 4 | 5 | |
|  | 14 | Feeling hopeless about the future | 1 | 2 | 3 | 4 | 5 | |
|  | 15 | Feeling so restless you couldn’t sit still | 1 | 2 | 3 | 4 | 5 | |
|  | 16 | Feeling weak in parts of your body | 1 | 2 | 3 | 4 | 5 | |
|  | 17 | Thoughts of ending your life | 1 | 2 | 3 | 4 | 5 | |
|  | 18 | Feeling fearful | 1 | 2 | 3 | 4 | 5 | |
|  |  |  |  |  |  |  |  | |

Clinical data

*Below are some questions about your cancer:*

1. What was your childhood cancer diagnosis?


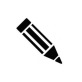


………………………………………………………

1. How old were you (in years) when you were diagnosed with cancer?


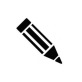


………………………………………………………

1. **What treatments did you receive (multiple answers possible)?**

☐ Chemotherapy

☐ Radiotherapy

☐ Operation

☐ Bone marrow transplantation

1. **When did you complete your treatment(s)?**


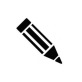


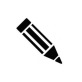
Years ago

Or in the year

1. **Do you still go for regular follow-up visits?**

☐ Yes

☐ No, the planned follow-up checks have been completed.

☐ No, I never went for follow-up examinations because of my cancer.

*With the following questions, we would like to find out from you whether you suffer from late effects. By late effects we mean physical or psychological problems that have arisen as a result of the disease and/or associated treatments.*

1. Do you suffer from physical and/or psychological late effects of cancer or related treatments?

☐ No

☐ Yes

- 1. **If yes, which ones?**


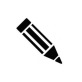


………………………………………………………………………………………………

………………………………………………………………………………………………

………………………………………………………………………………………………

1. Have you ever been diagnosed with a recurrence of your first cancer?

☐ No

☐ Yes

1. Did you have another cancer (e.g., leukemia, tumor, or skin cancer) after this first childhood cancer?


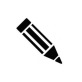
☐ No

☐ Yes, Diagnosis: ...………………


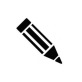


Month/year of diagnosis:

Information about yourself

*Finally, a few details about yourself:*

1. How old are you?


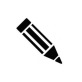


………………

1. Gender

☐ female ☐ male

1. What is your nationality (multiple answers possible)?


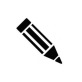


☐ Swiss ☐ other nationality: ……

1. Do you live in a partnership (multiple answers possible)?

☐ Single

☐ Relationship/concubinage Since (years ____________)

☐ Married Since (years ____________)

☐ Widowed Since (years ____________)

☐ Separated/divorced Since (years ____________)

1. Do you have children?

☐ Yes

☐ No

1. Please indicate your highest level of education completed:

| ☐ | None |
| --- | --- |
| ☐ | Up to a maximum of 7 years of compulsory school |
| ☐ | Compulsory school (real, secondary, district, orientation school, pro, lower secondary school, special school) |
| ☐ | 1-year pre-apprenticeship, 1-year general education school, 10th school year, 1-year vocational school, household apprenticeship year, language school (at least 1 year) with final certificate, social year, bridge programs or similar training |
| ☐ | 2- to 3-year diploma high school, traffic school, specialized high school FMS or similar education |
| ☐ | Basic vocational training (apprenticeship, 2- to 4-year vocational apprenticeship or full-time vocational school, trade diploma, apprenticeship workshop or similar training) |
| ☐ | Gymnasiale Maturität, Teacher Training Seminar (preparatory training for teachers of kindergarten, elementary school, manual work, home economics) |
| ☐ | Vocational or technical baccalaureate |
| ☐ | Higher technical and vocational training with federal certificate, federal diploma or master craftsman's diploma, higher technical school for technology (Technikerschule TS), for economics (HKG) or similar higher technical school (2 years full or 3 years part-time study) |
| ☐ | Higher technical college (predecessor of universities of applied sciences, e.g. HTL, HWV, HFG, HFS) including postgraduate diplomas (3 years of full- or 4 years of part-time studies) |
| ☐ | Bachelor (university, ETH, university of applied sciences, university of education) |
| ☐ | Master's degree, licentiate, diploma, state examination, postgraduate degree (university, ETH, university of applied sciences, university of teacher education) |
| ☐ | Doctorate, Habilitation |

1. What is your current professional situation on the labor market (multiple answers possible)?

☐Employed

Number of hours per week: _______Hours

☐ Looking for a job (registered with unemployment insurance or not)

☐ In training (school, study, apprenticeship)

☐ Housewife/Man

☐ Invalid or partially disabled (e.g. IV pensioner)

☐ Other situation without gainful employment

| This page provides space for you to send us your questions, comments, criticisms and suggestions about the study. We welcome your feedback. |
| --- |
| 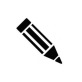 |

**Thank you for your participation!**

**We would like to thank you very much for your help!**

 [
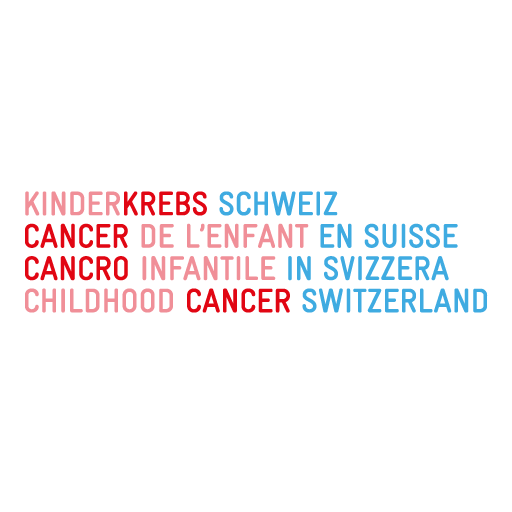
](https://www.google.ch/url?sa=i&rct=j&q=&esrc=s&source=images&cd=&cad=rja&uact=8&ved=0ahUKEwij5Jjmr_7UAhWCOBQKHexjBUQQjRwIBw&url=https://www.kinderkrebs-schweiz.ch/&psig=AFQjCNGdZ77VL2RjQOUYh06uSepHhCtyhg&ust=1499764356830342)
